# Incidence and Risk Factors of Intraocular Foreign Body-Related Endophthalmitis in Southern China

**DOI:** 10.1155/2018/8959108

**Published:** 2018-09-30

**Authors:** Fang Duan, Zhaohui Yuan, Jingyu Liao, Yongxin Zheng, Yao Yang, Xiaofeng Lin

**Affiliations:** Zhongshan Ophthalmic Center, State Key Laboratory of Ophthalmology, Sun Yat-sen University, Guangzhou 510060, China

## Abstract

**Purpose:**

To investigate the incidence and risk factors of intraocular foreign body- (IOFB-) related endophthalmitis.

**Methods:**

A total of 1701 patients diagnosed with IOFB between January 1, 2005 and June 30, 2015 were included. Two groups of patients were defined according to the presence or absence of endophthalmitis, and a comparison of personal information, IOFB characteristics, and wound location were performed.

**Results:**

In total, 279 patients (16.4%) developed endophthalmitis, older age (*P*=0.01) was a risk factor. IOFBs retained in the crystal lens or wall of the eyeball conferred lower risks (*P*=0.01 and 0.04, respectively) compared to the vitreous chamber. The coexistence of different IOFB types and plant IOFBs conferred higher risks (*P*=0.02 and 0.03, respectively), while glass/plastic IOFBs conferred a lower risk (*P*=0.03) compared to metallic IOFBs.

**Conclusions:**

Age, IOFB locations, and types were related to development of endophthalmitis, while IOFB number, size, or timing of primary repairs was not related.

## 1. Introduction

Retained intraocular foreign body (IOFB) occurs in 18%–41% of ocular trauma cases, leading to a wide range of ocular pathologies and vision outcomes [1]. One of IOFBs' potential complications, endophthalmitis, is of particular concern due to its tendency towards rapid vision loss, even blindness. Endophthalmitis has also been included in the Ocular Trauma Score as an indicator for poor visual prognosis [2]. The prevalence of traumatic endophthalmitis has been reported to occur in approximately 4% to 8% of cases and may be higher at 6.9% to 30% in IOFB injuries [3]. In addition, IOFB is reported to be present in 43% of eyes diagnosed with traumatic endophthalmitis [4].

IOFBs as risk factors for traumatic endophthalmitis were reported by many studies. However, the specific characteristic of IOFB which was associated with endophthalmitis development was less known. Several studies reported that nonmetallic IOFBs had a higher risk of endophthalmitis compared to metallic IOFBs [5, 6]. However, this trend was not shown in another study [7]. The data involve very small numbers of patients in these studies. Additionally, limited information is available in Asian populations regarding IOFB and associated endophthalmitis. Thus, the purposes of our study were to (1) assess the incidence of IOFB-related endophthalmitis in a Chinese population and (2) relate the IOFB characteristics, including number, type, size, location, and wound location and time of primary repair, to the development of endophthalmitis.

## 2. Method

### 2.1. Population

A total of 1701 patients admitted to Zhongshan Ophthalmic Center (ZOC), Guangzhou, China, and who were diagnosed with IOFBs between January 1, 2005 and June 30, 2015 were included in the current study. All electronic medical records were stored in a centralized hospital medical database and reviewed retrospectively for all the study participants. This study was performed in compliance with the principles of the Declaration of Helsinki and was approved by the Institutional Ethics Committee of Zhongshan Ophthalmic Center, Sun Yat-sen University.

### 2.2. Procedures

All patients underwent a detailed ophthalmic examination by a senior ophthalmologist upon admission. Generally, patients received immediate primary wound closing and conventional intravenous antibiotics injection and IOFB removal surgery as soon as possible. The antibiotics mainly included cefuroxime, levofloxacin, and clindamycin alone or in combination. The presence and characteristics of IOFBs were assessed by the ophthalmologist and recorded in the electronic medical record system. The number of IOFBs was counted and recorded accordingly. The type of IOFB was recorded as the name of the pathogenic object and classified as metallic, glass/plastic, eyelash, plant, coexistence (≥2 coexistent IOFB types) and other types in this study for analysis. The location of the IOFB was determined by the ophthalmologist as the deepest location of the retained IOFB from the cornea and was further classified into 5 groups: wall of the eyeball, anterior chamber, crystalline lens, vitreous cavity, and perforation. Specifically, an IOFB that presented an exit wound in the posterior global wall and partly stayed in vitreous cavity was classified into the perforation group. The size of the IOFB was recorded as the length (mm) in three dimensions: length × width × height. In addition, the longest diameter, regardless of the number of IOFBs, was used to categorize the IOFB into 4 size groups: <3 mm, 3–5 mm, >5–10 mm, and >10 mm. Wound location was also assessed and classified into 3 groups: cornea, sclera and corneosclera, the last which indicated a wound involving both the cornea and sclera.

The following information was also record in the medical record and obtained for analysis: personal information, including age and gender. The primary repair time was calculated by time of ocular injury and time of primary wound repair surgery. Patients were dichotomized into ≤24 h and >24 h groups based on their primary repair times. Diagnosis of endophthalmitis was based on clinical manifestations, and presence or absence of endophthalmitis before discharge, as the study outcome.

### 2.3. Statistical Method

A group *t*-test and chi-square test were used to compare the patient characteristics between patients with IOFB who developed endophthalmitis and those who did not. Univariate logistic regression was used to assess the association between individual IOFB characteristics and the development of endophthalmitis. And only IOFB characteristics with a *P* value of less than 0.2 in the univariate analysis were included in the multiple regression analysis. Reference groups were chosen based on subgroup frequency and clinical significance. For patients with binocular IOFBs, the more severe eye was selected into the analysis. *P* values of <0.05 were considered to be statistically significant.

## 3. Results

Of the 1701 consecutive patients with IOFBs in our study, 279 (16.4%) were clinically diagnosed with endophthalmitis, and 74 had culture-proven endophthalmitis. No patient had an enucleation/evisceration on the first surgery. The mean age of the study participants was 31.7 ± 12.5 years, and only 7.88% were female. The majority of patients had only 1 IOFB (91.7%), and the most common type and location of the IOFB was metal (78.5%) and vitreous cavity (74.3%), respectively. The data of the IOFB size were unavailable for 165 patients. For the remaining 1536 patients, the percentages of patients within the <3 mm, 3–5 mm, >5–10 mm, and >10 mm groups were 38.0%, 29.1%, 21.2%, and 11.7%, respectively. The percentages of patients with primary repair within 24 hours of injury and more than 24 hours of injury were similar (55.9% vs. 44.1%, respectively). The cornea was the most common wound site (67.3%). Details of the IOFB characteristics in the current study can be found in Table 1. Specifically, the prevalence of endophthalmitis in different IOFB material group is shown in Table 2.

Comparison of IOFB characteristics between patients with and without endophthalmitis is shown in Table 1. Age, IOFB location, and type were significantly different between these two groups (*P*=0.02, 0.004, and 0.008, respectively), while the distributions by gender, IOFB number, size, and primary repair time were similar. The associations between intraocular foreign body characteristics and endophthalmitis were analyzed by logistic regression.  Table 3 shows that older age (*P*=0.01) was a risk factor for endophthalmitis, even after adjusting for the IOFB characteristics. IOFBs retained in the crystal lens (*P*=0.01) or the wall of the eyeball (*P*=0.04) conferred a lower risk of endophthalmitis compared to IOFBs retained in the vitreous chamber. In addition, plant IOFBs (*P*=0.03) and the coexistence of different IOFB types (*P*=0.02) conferred a higher endophthalmitis risk, while glass/plastic IOFBs (*P*=0.03) conferred a lower risk compared to metallic IOFBs. Gender, time of primary repair, and wound location, as well as IOFB number or size, were not significantly related to the risk of endophthalmitis.

## 4. Discussion

This study included all patients with IOFB-related ocular injuries admitted to a tertiary hospital over 10 years and reported the incidence of endophthalmitis and its associations with potential IOFB characteristic factors. Strengths included a large sample size and the availability of multiple IOFB characteristics.

We reported an endophthalmitis incidence of 16.4% in this IOFB-related ocular injury patient group, which is similar to that observed in a previous study in China (16.76%) [8]. This rate is higher than reported in other populations, such as those in Saudi Arabia, America, and Iran (5%–13%) [9–11]. Differences in ethnicity, patient characteristics, and treatment therapy might explain the variance in endophthalmitis incidence across different regions. In addition, the observed higher incidence in our study could be because the IOFB patients admitted to our hospital were more severe and late-delivered, given that ZOC received patients from all over the country and mostly severe cases that could not be treated at local hospitals.

Our finding is consistent with the universal characteristic of IOFB distribution in previous studies, including male predominance, metallic IOFB as the most prevalent type, and the majority of IOFBs reaching the posterior segment of the eye [12–14]. The risk of endophthalmitis in the presence of IOFB was reported to increase with age [7], which is also found in our study. The reason was suggested to be the correlation between delay in trauma repair with age [7, 10]; however, we found that age was significant even after adjusting for time of primary repair. Slower wound healing and weaker immune function with aging could be possible explanations. According to a literature review by Kuhn et al., multiple IOFBs were found in 8–25% of the injuries, and the average size of an IOFB was 3.5 mm [15]. Our result is consistent with this report (8.35% and 4.9 mm), while neither IOFB number nor size was related to the development of endophthalmitis. Similar results were reported by previous studies [16], and IOFB size was suggested to be more related to postsurgical vision acuity [12, 13, 16].

Whether IOFB material was related to endophthalmitis risk has not been determined to date. A number of studies showed that the nature of the IOFB will not affect the development of endophthalmitis [7, 16]. We found a higher risk in plant IOFBs and a lower risk in glass/plastic IOFBs compared to metallic IOFBs. Several studies reported that organic IOFBs and wood and soil contamination greatly increase the risk of endophthalmitis [6, 17], which is consistent with our results. The probable reason is that plant IOFBs are more likely to carry microorganisms leading to ocular inflammation, while glass and plastic IOFBs are inert and thus less likely to induce endophthalmitis.

In addition, the coexistence of different types of IOFBs was significantly related to endophthalmitis in our study. Due to the small sample size in this subgroup (22 cases, 1.29%), this association needs further investigation.

Disruption of the crystalline lens was consistently reported to be a risk factor for endophthalmitis, with the reported incidence of posttraumatic endophthalmitis being considerably higher in cases with lens disruptions than that in those without (18% vs. 1%, respectively) [18, 19]. Leakage of lens particles inducing the inflammation process and the slower aqueous humor flow associated with lens rupture might explain the higher endophthalmitis risk. However, our study found a lower risk of crystalline lens-retained IOFBs compared to vitreous cavity-retained IOFBs. The reason could be that IOFBs embedded within the lens are not always accompanied by lens rupture, and the inert environment inside the lens could protect it from infection. Similarly, IOFBs in the wall of the eyeball were also found to confer a lower endophthalmitis risk, which could be explained by the inert environment inside the ocular wall and its isolation from the ocular immune system. In addition, associations between wound location and endophthalmitis remain a big area of debate. Zhang and Copper suggested that scleral wounds conferred a lower risk of endophthalmitis than corneal wounds, and Duch-Samper reported the opposite finding [20–22]. We found no significant difference in the risk among 3 trauma location groups, which is consistent with Dehghani's finding [23].

Limitations of this study included its retrospective nature and small sample size in certain subgroups. Visual acuity prognosis is an important aspect of IOFB-related injuries and endophthalmitis, but it was not assessed in this study due to the difficulty of patient follow-up in large tertiary hospitals. Nevertheless, our study provides valid data for understanding the association between IOFB characteristics and endophthalmitis.

In conclusion, our study observed a high incidence of IOFB-related endophthalmitis in this Chinese patient population. Age, IOFB location, and type were related to the development of endophthalmitis and should be taken into consideration in clinical practice.

## Figures and Tables

**Table 1:** Comparison of study participants with and without endophthalmitis.

Characteristics	Total (*n*=1701)	Endophthalmitis (*n*=279)	Nonendophthalmitis (*n*=1422)	*P* value
*Age, years*	31.7 ± 12.5	33.2 ± 13.9	31.4 ± 12.2	0.02
*Gender, female*	134 (7.88%)	20 (7.17%)	114 (8.02%)	0.63
*No. of IOFBs*				0.74
1	1559 (91.7%)	259 (92.8%)	1300 (91.4%)	
2	92 (5.41%)	13 (4.66%)	79 (5.56%)	
≥3	50 (2.94%)	7 (2.51%)	43 (3.02%)	
*Timing of primary repair*				0.25
≤24 h	950 (55.9%)	147 (52.7%)	803 (56.5%)	
>24 h	751 (44.1%)	132 (47.3%)	619 (43.5%)	
*Site of laceration*				0.20
Cornea	1145 (67.3%)	200 (71.7%)	945 (66.5%)	
Sclera	361 (21.2%)	49 (17.6%)	312 (21.9%)	
Corneosclera	195 (11.5%)	30 (10.7%)	165 (11.6%)	
*IOFB location*				0.004
Wall of eyeball	115 (6.76%)	11 (3.94%)	104 (7.31%)	
Anterior chamber	170 (9.99%)	30 (10.8%)	140 (9.85%)	
Crystalline lens	64 (3.76%)	2 (0.72%)	62 (4.36%)	
Vitreous cavity	1263 (74.3%)	225 (80.7%)	1038 (73.0%)	
Perforation	89 (5.23%)	11 (3.94%)	78 (5.49%)	
*Material of IOFB*				0.008
Metal	1335 (78.5%)	218 (78.1%)	1117 (78.6%)	
Glass/plastic	76 (4.47%)	4 (1.43%)	72 (5.06%)	
Eyelash	60 (3.53%)	11 (3.94%)	49 (3.45)	
Plant	26 (1.53%)	7 (2.51%)	19 (1.34%)	
Other	182 (10.7%)	31 (11.1%)	151 (10.62)	
Coexistence	22 (1.29%)	8 (2.87%)	14 (0.98%)	
*Maximum size of IOFB*				0.55
<3	583 (38.0%)	91 (35.3%)	492 (38.5%)	
≥3 to 5	448 (29.1%)	79 (30.6%)	369 (28.9%)	
>5 to 10	325 (21.2%)	61 (23.6%)	264 (20.7%)	
>10	180 (11.7%)	27 (10.5%)	153 (12.0%)	

IOFB: intraocular foreign body.

**Table 2:** The prevalence of endophthalmitis in different IOFB material group.

Material of IOFB	Total	Endophthalmitis	Prevalence (%)
Metal	1335	218	16.3
Glass/plastic	76	4	5.3
Eyelash	60	11	18.3
Plant	26	7	26.9
Other	182	31	17.0

**Table 3:** Associations between intraocular foreign body characteristics and endophthalmitis by logistic regression.

Factors	Univariate regression		Multiple regression	
	Regression coefficient mean (95% CI)	*P* value	Regression coefficient mean (95% CI)	*P* value
*Age*	0.01 (0.00 to 0.02)	0.02	0.01 (0.00 to 0.02)	0.01
*Gender*	−0.12 (−0.61 to 0.37)	0.63	—	—
*No. of IOFB*			—	—
1	Reference			
≥2	−0.19 (−0.69 to 0.30)	0.44		
*Timing of primary repair*			—	—
<24 h	Reference			
≥24 h	0.15 (−0.10 to 0.41)	0.25		
*Site of laceration*				
Cornea	Reference		Reference	
Sclera	−0.30 (−0.64 to 0.04)	0.08	−0.33 (−0.68 to 0.02)	0.07
Corneosclera	−0.15 (−0.57 to 0.27)	0.48	−0.08 (−0.50 to 0.35)	0.73
*IOFB location*				
Wall of eyeball	−0.72 (−1.36 to −0.08)	0.03	−0.70 (−1.37 to −0.04)	0.04
Anterior chamber	−0.01 (−0.43 to 0.41)	0.96	−0.07 (−0.54 to 0.40)	0.78
Crystalline lens	−1.91 (−3.32 to −0.49)	0.01	−1.98 (−3.41 to −0.56)	0.01
Vitreous cavity	Reference		Reference	
Perforation	−0.43 (−1.08 to 0.22)	0.19	−0.37 (−1.04 to 0.29)	0.27
*Material of IOFB*				
Metal	Reference		Reference	
Glass/plastic	−1.26 (−2.27 to −0.24)	0.02	−1.14 (−2.17 to −0.10)	0.03
Eyelash	0.14 (−0.53 to 0.81)	0.68	0.25 (−0.48 to 0.97)	0.51
Plant	0.64 (−0.24 to 1.51)	0.16	1.04 (0.11 to 1.97)	0.03
Else	0.05 (−0.36 to 0.46)	0.81	0.13 (−0.30 to 0.55)	0.56
Coexistence	1.07 (0.19 to 1.95)	0.02	1.10 (0.21 to 1.99)	0.02
*Maximum Size of IOFB*			—	—
<3	−0.15 (−0.48 to 0.18)	0.39		
≥3 to 5	Reference			
≥5 to 10	0.08 (−0.29 to 0.45)	0.69		
≥10	−0.19 (−0.67 to 0.28)	0.43		

IOFB: intraocular foreign body.

## Data Availability

All the data used to support the findings of this study are included within the article and are available from corresponding author by a reasonable request.
